# Evaluating the feasibility, sensitivity, and specificity of next-generation molecular methods for pleural infection diagnosis

**DOI:** 10.1128/spectrum.01960-24

**Published:** 2025-01-15

**Authors:** Peter T. Bell, Timothy Baird, John Goddard, Olusola S. Olagoke, Andrew Burke, Shradha Subedi, Tiana R. Davey, James Anderson, Derek S. Sarovich, Erin P. Price

**Affiliations:** 1 Department of Respiratory Medicine, Sunshine Coast University Hospital, Birtinya, Queensland, Australia; 2 Sunshine Coast Health Institute, Birtinya, Queensland, Australia; 3 Faculty of Medicine, University of Queensland, Herston, Queensland, Australia; 4 Centre for Bioinnovation, University of the Sunshine Coast, Sippy Downs, Queensland, Australia; 5 School of Medicine and Dentistry, Griffith University, Sunshine Coast, Queensland, Australia; 6 Department of Respiratory and Thoracic Medicine, The Prince Charles Hospital, Chermside, Queensland, Australia; 7 Department of Infectious Diseases, Sunshine Coast University Hospital, Birtinya, Queensland, Australia; ICON plc, London, United Kingdom

**Keywords:** empyema, metagenomics, high-throughput sequencing, parapneumonic effusion, pleural infection, microbiome

## Abstract

Pleural infections are common and associated with substantial healthcare costs, morbidity, and mortality. Accurate diagnosis remains challenging due to low culture positivity rates, frequent polymicrobial involvement, and non-specific diagnostic biomarkers. Here, we undertook a prospective study examining the feasibility and performance of molecular methods for diagnosing suspected pleural infection. We prospectively characterized 26 consecutive clinically suspected pleural infections, and 10 consecutive patients with suspected non-infective pleural effusions, using shotgun metagenomics, bacterial metataxonomics, quantitative PCR, and conventional culture. Molecular methods exhibited excellent diagnostic performance, with each method identifying 54% (14 out of 26) positive cases among the pleural infection cohort, versus 38% (10 out of 26) with culture. Metagenomics and bacterial metataxonomics unveiled complex polymicrobial infections that were not captured by culture. Dominant microbes included streptococci (*Streptococcus intermedius*, *Streptococcus pyogenes*, and *Streptococcus mitis*), *Prevotella* spp. (*Prevotella oris* and *Prevotella pleuritidis*), staphylococci (*S. aureus* and *S. saprophyticus*), and *Klebsiella pneumoniae*. However, we encountered challenges that complicated pleural infection interpretation, including: (i) uncertainties regarding microbial pathogenicity and the impact of prior antibiotic therapy on diagnostic performance; (ii) lack of a clinical diagnostic gold-standard for molecular performance comparisons; (iii) potential microbial contamination during specimen collection or processing; and (iv) difficulties distinguishing background microbial noise from true microbial signal in low-biomass specimens. This pilot study demonstrates the potential utility and value of molecular methods in diagnosing pleural infection and highlights key concepts and challenges that should be addressed when designing larger prospective trials.

**IMPORTANCE:**

Confident pleural infection diagnosis is often challenging due to low culture positivity rates, frequent polymicrobial involvement, and non-specific diagnostic biomarkers. Limitations of conventional diagnostic tests result in prolonged and inappropriately broad-spectrum antimicrobial use, encouraging antimicrobial resistance and leading to avoidable adverse effects. Here, we demonstrate the feasibility, utility, and challenges associated with the use of culture-independent molecular techniques for accurate pleural infection diagnosis in a real-world clinical setting. These data will help to inform the design of larger prospective clinical trials and identify potential obstacles to be overcome before next-generation sequencing technologies can be integrated into routine clinical practice.

## INTRODUCTION

Pleural infection is a complex and heterogeneous clinical entity associated with prolonged hospitalization, significant healthcare costs, and high morbidity and mortality (1–4). Challenges in clinical management arise due to insensitivities in conventional microbiologic diagnostics (culture-positive rates of ~40–60%) (5), resulting in prolonged and potentially inappropriate broad-spectrum antimicrobial use. Culture-based diagnostic sensitivity is poor due to multiple factors, including empiric antibiotic therapy prior to pleural sampling, low pathogen concentration in pleural fluid, and difficulties in cultivating fastidious microorganisms (4, 6). These limitations have obscured the characterization of the pleural “microbiome” (i.e., the entire microbial community), hampering clinical progress (7).

Molecular diagnostics enable culture-independent microbial identification from clinical specimens (8). Such techniques include shotgun metagenomics, metataxonomics, and quantitative PCR (qPCR), each having unique capabilities and drawbacks. Shotgun metagenomics involves total microbiome DNA sequencing of a sample, allowing strain-level detection of bacteria, archaea, fungi, parasites, and DNA viruses, along with their putative functions (e.g., antimicrobial resistance [AMR], virulence). However, metagenomics overlooks RNA viruses and cannot readily differentiate living from dead microbes. Metataxonomics involves next-generation amplicon sequencing of conserved bacterial (16S ribosomal RNA [rRNA]) or fungal internal transcribed spacer (ITS) regions to characterize bacterial or fungal consortia, respectively. However, metataxonomic methods typically only resolve microbiota to the genus level, and cannot be used for relative taxon quantification due to highly variable 16S rRNA/ITS copy numbers among species. Finally, qPCR is an inexpensive, same-day diagnostic method, with broad resolution ranging from strain to domain level; however, this method is typically limited to *a priori* target taxa (9, 10).

Several recent studies have applied culture-independent techniques to characterize pleural infections. Collectively, this work has revealed a pleural microbiome that is frequently polymicrobial and often characterized by a diverse range of classical pathogens and potential bystander microbes (5, 11–19). Earlier investigations implemented bacterial metataxonomics to characterize the bacterial microbiome (5, 11, 12, 18); however, three recent studies have implemented metagenomics to more comprehensively profile pleural microbiota (13, 16, 17). Xu et al. found bacterial dominance in 80 adult Chinese patients with pleural infections, most commonly *Streptococcus* and *Prevotella* spp., although fungi were occasionally identified (13). However, the authors only reported taxonomic findings to the genus level, limiting in-depth understanding of microbiome composition and diversity (13). Liu et al. reported on a single case study of viral pleurisy caused by Epstein-Barr virus, which was successfully treated with acyclovir (17). Finally, Liu et al. examined microbiomes of pleural and ascitic fluid in 92 specimens retrieved from 32 Chinese children, identifying *Klebsiella pneumoniae* as the dominant pathogen (*n* = 17), followed by *Escherichia coli* (*n* = 9), and *Acinetobacter baumannii* (*n* = 7); yeasts (*n* = 6) and several viruses (*n* = 33) were also identified (16). In all three studies, metagenomics yielded a higher pathogen-positive rate than conventional culture (13, 16, 17).

To expand knowledge of the pleural microbiome in other cohorts, we undertook a prospective, observational, single-center pilot study of 26 consecutive patients presenting with suspected pleural infection at an Australian hospital, and 10 consecutive patients lacking clinical suspicion of pleural infection. We compared the feasibility and diagnostic performance of microbe-enriched shotgun metagenomics, bacterial metataxonomics, panbacterial qPCR, and panfungal qPCR with conventional culture.

## RESULTS

### Study participants and clinical outcomes

Twenty-six participants (*n* = 7 female; median age = 67 years [range 20–95]) with suspected pleural infection had an exudative pleural effusion as defined by Light’s criteria (20). Participants were further divided into 16 Probable and 10 Possible pleural infections based on clinical criteria (Table 1; see Study Participants section). Twenty-four (92%) had loculated (complex) pleural effusions on ultrasound and/or computed tomography imaging, with 20 (77%) also having associated consolidation at the time of cross-sectional imaging. Twenty-five (96%) underwent intercostal catheter insertion, whereas 1 underwent diagnostic/therapeutic needle aspiration. All 26 patients received broad-spectrum antimicrobial therapy (Table 2). Intrapleural enzyme therapy (5 mg DNase and 10 mg tissue plasminogen activator) was administered in 17 participants (65%), and 4 (15%) required surgical decortication (Table 1; Table S1). Thirty-day mortality was 12% (*n* = 3).

**TABLE 1:** Summary of clinical, demographic and outcome data^*a*^

	Probable pleural infection	Possible pleural infection	Unlikely pleural infection (control)
Clinical/demographics			
Number of participants in group, *n*	16	10	10
Age in years (median, range)	63 (20–90)	71 (44–95)	78 (41–87)
Female sex, *n* (%)	3 (19)	4 (40)	2 (20)
Immunosuppression, *n* (%)	0 (0)	3 (30)	1 (10)
Fever, *n* (%)	15 (94)	8 (80)	0 (0)
ICU admission, *n* (%)	5 (31)	1 (10)	0 (0)
Biochemical			
Serum white cell count, cells ×10^9^/L, median (range)	15.9 (10.2–33.6)	10.6 (8.4–20.1)	8.3 (4.3–15.2)
Serum C-reactive protein, mg/L, median (range)	237 (39–547)	127 (39–270)	56 (5–124)
Serum procalcitonin, µg/L, median (range)	0.9 (0.1–9.6)	0.2 (0.1–4.3)	0.2 (0.1–0.3)
Serum LDH, unit/L, median (range)	222 (108–296)	289 (169–477)	208 (160–391)
Serum protein, g/L, median (range)	61 (49–76)	60 (49–71)	62 (45–72)
Fluid LDH, unit/L, median (range)	1,615 (173–17,400)	536 (280–1,200)	239 (81–672)
Fluid protein, g/L, median (range)	47 (28–58)	39 (23–53)	43 (37–46)
Fluid pH, median (range)	6.9 (6.6–7.2)	7.3 (7.3–7.5)	7.4 (7.3–7.5)
Fluid glucose, mmol/L, median (range)	0.3 (0.3–5.9)	5.0 (3.3–11.9)	6.3 (4.6–10.0)
Imaging			
Consolidation, *n* (%)	12 (75)	7 (70)	0 (0)
Complex effusion appearance, *n* (%)	15 (94)	9 (90)	0 (0)
Outcome data			
IPE, *n* (%)	12 (75)	5 (50)	0 (0)
CTS, *n* (%)	3 (19)	1 (10)	0 (0)
30-day mortality, *n* (%)	2 (13)	1 (10)	Not determined

^*a*^ Abbreviations: CTS, cardiothoracic surgical decortication; ICU, intensive care unit; IPE, intrapleural enzyme therapy; LDH, lactate dehydrogenase.

**TABLE 2:** Antimicrobial therapy type and specimen collection time in participants diagnosed with pleural infection, along with detected antimicrobial resistance (AMR) loci^*a*^

Participant ID	Antibiotics administered	Time prior to PF collection	AMR genes^*b*^
Probable pleural infection			
SCHI0123-P	AMC, AZM, CRO, TZP	64 h	Nil
SCHI0125-P	AZM, CRO, TZP	22 h	Nil
SCHI0127-P	AZM, MXF	15 h	*erm*(*A*) (streptogramin B resistance, CLIr, ERYr, LINr)
SCHI0131-P	AZM, TZP	6 h	*cfxA3* (AMPr), *mef*(*A*) (ERYr, AZMr), *msr*(*D*) (AZMr, ERYr, TELr, streptogramin B resistance), *tet*(*32*) and *tet*(*O*) (DOXr, MINr, TETr)
SCHI0132-P	AZM, CRO, TZP	120 h	Nil
SCHI0133-P	CRO, TZP, VAN	48 h	Nil
SCHI0134-P	TZP	19 h	Nil
SCHI0135-P	TZP	7 h	Nil
SCHI0136-P	TZP	3 h	Nil
SCHI0140-P	AZM, TZP, VAN	8 h	Nil
SCHI0141-P	AMC (oral for 5 days), TZP (IV for 72 h)	72 h	Nil
SCHI0157-P	CAZ, DOX, metronidazole	13 h	Nil
SCHI0158-P	AMC, FEP, TZP	144 h	Nil
SCHI0163-P	AMC	24 h	*aph(6)-Id* (STRr)
SCHI0166-P	AMC, BPG, DOX	72 h	Nil
SCHI0172-P	AMC, TZP	36 h	*tet*(*M*) (DOXr, MINr, TETr)
Possible pleural infection			
SCHI0126-P	AMC	96 h	Nil
SCHI0128-P	CRO, TZP	112 h	Nil
SCHI0143-P	CAZ	21 days	Nil
SCHI0148-P	Nil	0 h	Nil
SCHI0149-P	Nil	0 h	Nil
SCHI0150-P	AZM, BPG, CRO	102 h	Nil
SCHI0159-P	AZM, BPG, CRO	10 days	Nil
SCHI0160-P	TZP	21 days	Nil
SCHI0162-P	AZM, BPG, CRO, TZP	10 days	Nil
SCHI0169-P	AMX (then AMC), DOX	94 h	Nil

^*a*^ Abbreviations: AMC, amoxicillin-clavulanate; AMR, antimicrobial resistance; AMX, amoxicillin; AZM, azithromycin; AZMr, azithromycin resistance; BPG, benzathine penicillin G; CAZ, ceftazidime; CLIr, clindamycin resistance; CRO, ceftriaxone; DOX, doxycycline; DOXr, doxycycline resistance; ERYr, erythromycin resistance; FEP, cefepime; IV, intravenous; LINr, lincomycin resistance; MINr, minocycline resistance; MXF, moxifloxacin; STRr, streptomycin resistance; TELr, telithromycin resistance; TETr, tetracycline resistance; TZP, piperacillin/tazobactam; VAN, vancomycin.

^*b*^ Identified from metagenomic data using ResFinder.

Of the 10 unlikely pleural infection subjects, 9 were diagnosed with malignant pleural effusion (MPE), and 1 with decompensated congestive heart failure (Table 1; Table S1). Thirty-day mortality was not recorded for this cohort.

### Conventional culture performance

Pathology lab testing returned 10 (38%) microbial culture-positive results from the suspected pleural infection cohorts, whereas only 6 (23%) were culture-positive from Research lab testing (Table 3); however, there was 100% overlap with the Pathology results. Unexpectedly, pleural fluid from two (20%) Unlikely infection participants, SCHI0152-C and SCHI0175-C, cultured *Candida parapsilosis* and *Moraxella osloensis*, respectively, in the Research lab. No microbes were cultured in the Unlikely infection cohort by the Pathology lab.

**TABLE 3:** Diagnostic findings across 26 Probable or Possible pleural infection-diagnosed participants and 10 controls diagnosed with a non-infection etiology^*a*^

Participant ID	Metagenomic result^*b*^	Bacterial metataxonomic result^*c*^	Culture result, Research Lab (WGS result)	Culture result, Pathology Lab (MALDI-TOF result)	Panbacterial qPCR^*d*^
Probable pleural infection					
SCHI0123-P	Polymicrobial (*Fusobacterium nucleatum*, *Fusobacterium hwasookii*, *Staphylococcus* phage Andhra, *Prevotella loescheii*, *Rothia mucilaginosa*, *Enterococcus faecium*, *Fusobacterium* sp. oral taxon 203)	Monomicrobial (*Fusobacterium*)	NG	NG	Yes (*C_T_* = 20.8)
SCHI0125-P	Polymicrobial (*Staphylococcus* phage Andhra, *Prevotella oris, Enterococcus faecium*, *Streptococcus mitis*, *Streptococcus anginosus*, ***Escherichia coli*^*f*^**, *Streptococcus pneumoniae, Fonsecaea pedrosoi*, *Candida tropicalis, Pyricularia pennisetigena*, ***Staphylococcus aureus^g^***)	Monomicrobial (*Prevotella*)	***Escherichia coli^e^*, *Staphylococcus saprophyticus^e^***	*Escherichia coli*	Yes (*C_T_* = 25.7)
SCHI0127-P	Polymicrobial (*Streptococcus pyogenes*, *Staphylococcus* phage Andhra, *Streptococcus dysgalactiae*, *Streptococcus agalactiae*, *Enterococcus faecium*, *Rothia mucilaginosa*)	Monomicrobial (*Streptococcus*)	Positive (not characterized)	*Streptococcus pyogenes*	Yes (*C_T_* = 27.5)
SCHI0131-P^*j*^	Polymicrobial (*Streptococcus mitis*, *Streptococcus anginosus*, *Streptococcus pneumoniae*, *Streptococcus* sp. oral taxon 431*, Streptococcus oralis*, *Streptococcus salivarius*, *Gemella haemolysans*, *Streptococcus pseudopneumoniae*, *Streptococcus* sp. ChDC B345, *Streptococcus gordonii*, *Streptococcus parasanguinis, Streptococcus cristatus, Streptococcus* sp. HSISM1*, Streptococcus australis, Streptococcus* sp. oral taxon 064*, Streptococcus* sp. FDAARGOS_192*, Streptococcus intermedius, Streptococcus* sp. A12*, Veillonella atypica*, *Actinomyces odontolyticus*, *Streptococcus sanguinis*, *Gemella sanguinis*, *Streptococcus thermophilus*, *Capnocytophaga leadbetteri*, *Streptococcus equinus*, *Streptococcus viridans*, *Rothia dentocariosa*, *Capnocytophaga gingivalis*, *Veillonella parvula, Rothia mucilaginosa, Streptococcus vestibularis, Streptococcus* sp. HSISS2*, Prevotella jejuni, Streptococcus constellatus, Streptococcus* sp. I-P16*, Streptococcus* sp. I-G2*, Veillonella dispar, Bifidobacterium longum, Streptococcus* sp. HSISS3*, Gemella morbillorum*, *Haemophilus influenzae*, *Streptococcus* sp. HSISS1*, Atopobium parvulum*, *Streptococcus dysgalactiae*, *Streptococcus pyogenes*, *Streptococcus agalactiae*, *Staphylococcus aureus, Prevotella salivae, Prevotella melaninogenica, Tannerella* sp. oral taxon HOT-286*, Actinomyces* sp. oral taxon 171*, Actinomyces oris, Capnocytophaga sputigena, Haemophilus parainfluenzae, Neisseria subflava, Campylobacter showae, Streptococcus* phage SM1*, Streptococcus mutans, Streptococcus sobrinus, Streptococcus acidominimus, Lactococcus lactis, Lachnospiraceae* bacterium oral taxon 500*, Actinomyces naeslundii, Actinomyces viscosus, Capnocytophaga* sp. oral taxon 878, *Neisseria elongata*, *Lautropia mirabilis*, *Fusobacterium periodonticum*, *Fusobacterium nucleatum*, *Streptococcus ratti*, >**70 additional taxa^*h*^**)	Polymicrobial (*Streptococcus*, *Streptococcus anginosus*, *Granulicatella*, *Streptococcus salivarius*, *Gemella*, *Prevotella jejuni*, *Veillonella*, *Streptococcus constellatus*, *Prevotella melaninogenica*; *Neisseria perflava*, *Neisseria*, *Fusobacterium periodonticum*, *Prevotella salivae*, *Haemophilus influenzae*, *Leptotrichia*, *Capnocytophaga gingivalis*, *Capnocytophaga leadbetteri*, *Schaalia odontolytica*, *Neisseria elongata*, *Atopobium*, *Fusobacterium*, *Porphyromonas*, *Bergeyella*, *Leptotrichia hongkongensis*, *Oribacterium*, *Lautropia*, *Oribacterium*, *Selenomonas artemidis*, *Stomatobaculum*, *Haemophilus*, *Prevotella shahii*, *Rothia*)	***Pichia kudriavzevii^e^***	*Pichia kudriavzveii*; enteric and mixed anaerobic bacteria	Yes (*C_T_* = 17.7)
SCHI0132-P	Monomicrobial (***Staphylococcus aureus^g^***)	Monomicrobial (***Streptococcus^i^***)	Positive (not characterized)	Coagulase-negative *Staphylococcus* sp.	No (*C_T_* = 33.5)
SCHI0133-P	ND	ND	NG	NG	No (*C_T_* = 33.9)
SCHI0134-P	Monomicrobial (*Klebsiella pneumoniae*)	Monomicrobial (*Klebsiella*)	NG	*Klebsiella pneumoniae*	Yes (*C_T_* = 23.5)
SCHI0135-P	ND	ND	NG	NG	No (*C_T_* = 34.5)
SCHI0136-P	Monomicrobial (*Streptococcus intermedius*)	ND	NG	*Streptococcus intermedius*	Yes (*C_T_* = 27.5)
SCHI0140-P	Polymicrobial (*Streptococcus pyogenes*, *Streptococcus dysgalactiae*, *Candida dubliniensis*)	Polymicrobial (*Streptococcus, Streptococcus pyogenes*)	NG	*Streptococcus pyogenes*, *Staphylococcus aureus*	Yes (*C_T_* = 25.2)
SCHI0141-P	ND	ND	NG	NG	No (*C_T_* = 33.5)
SCHI0157-P	Monomicrobial (*Streptococcus intermedius*)	Monomicrobial (*Streptococcus*)	NG	*Streptococcus intermedius*	Yes (*C_T_* = 24.2)
SCHI0158-P	ND	Polymicrobial (*Fusobacterium*, *Parvimonas*, *Eubacterium*)	NG	NG	Yes (*C_T_* = 29.2)
SCHI0163-P	Monomicrobial (*Haemophilus parainfluenzae*)	Polymicrobial (*Haemophilus*, *Novosphingobium*)	NG	NG	Yes (*C_T_* = 23.5)
SCHI0166-P	Monomicrobial (*Escherichia coli*)	Monomicrobial (*Escherichia-Shigella*)	Positive (lactose fermenter not further characterized)	*Escherichia coli*	Yes (*C_T_* = 21.5)
SCHI0172-P	Polymicrobial (*Prevotella pleuritidis*, *Fusobacterium nucleatum*, *Prevotella enoeca*, *Prevotella intermedia*, *Hallela seregens*, *Porphyromonas gingivalis*, *Tannerella forsythia*, *Prevotella buccae*, *Prevotella oris*, *Fusobacterium* sp. oral taxon 203, *Prevotella nigrescens*, *Prevotella denticola*, *Streptococcus pyogenes*, *Prevotella veroralis*, *Fusobacterium hwasookii*, *Prevotella aurantiaca, Bacteroides fragilis*, *Bacteroides heparinolyticus*, *Prevotella salivae*, *Prevotella fusca*, *Prevotella pallens*, *Prevotella nanceiensis*, *Prevotella jejuni*, *Prevotella oulorum*, *Prevotella maculosa*, *Prevotella bivia*, *Prevotella melaninogenica*, *Prevotella micans*, *Prevotella amnii*, *Prevotella conceptionensis*, *Enterococcus faecium*)	Polymicrobial (*Enterococcus*, *Prevotella pleuritidis*)	Positive (not characterized)	*Fusobacterium nucleatum*	Yes (*C_T_* = 20.3)
Possible pleural infection					
SCHI0126-P	ND	ND	NG	NG	No (*C_T_* = 33.9)
SCHI0128-P	Polymicrobial (*Klebsiella pneumoniae*, *Staphylococcus* phage Andhra, *Mycoplasma hyorhinis*, *Carnobacterium divergens*, *Actinomyces odontolyticus*, *Rothia mucilaginosa*, *Streptococcus mitis*, *Haemophilus parainfluenzae*, *Enterococcus faecium*, *Streptococcus parasanguinis*, *Staphylococcus aureus*)	ND	NG	NG	No (*C_T_* = 35.3)
SCHI0143-P	Monomicrobial (*Staphylococcus saprophyticus*)	Monomicrobial (***Streptococcus^i^***)	NG	NG	Yes (*C_T_* = 30.1)
SCHI0148-P	ND	ND	NG	NG	No (*C_T_* = 34.6)
SCHI0149-P	ND	ND	NG	NG	No (*C_T_* = 31.3)
SCHI0150-P	ND	Monomicrobial (*Haemophilus*)	NG	NG	Yes (*C_T_* = 28.1)
SCHI0159-P	ND	ND	NG	NG	No (*C_T_* = 32.6)
SCHI0160-P	ND	ND	NG	NG	No (*C_T_* = 32.3)
SCHI0162-P	ND	ND	NG	NG	No (*C_T_* = 31.2)
SCHI0169-P	ND	ND	NG	NG	No (*C_T_* = 33.2)
Unlikely pleural infection					
SCHI0151-C	ND	ND	NG	NG	No (*C_T_* = 32.7)
SCHI0152-C	ND	Monomicrobial (*Escherichia-Shigella*)	***Candida parapsilosis^e^***	NG	No (*C_T_* = 35.7)
SCHI0161-C	ND	ND	NG	NG	No (*C_T_* = 36.7)
SCHI0164-C	ND	ND	NG	NG	No (*C_T_* = 35.9)
SCHI0165-C	ND	ND	NG	NG	No (*C_T_* = 32.3)
SCHI0168-C	ND	ND	NG	NG	No (*C_T_* = 32.7)
SCHI0170-C	ND	ND	NG	NG	No (*C_T_* = 38.7)
SCHI0173-C	Monomicrobial (adeno-associated dependoparvovirus A)	Polymicrobial (*Cloacibacterium*, *Corynebacterium*)	NG	NG	No (*C_T_* = 31.3)
SCHI0174-C	ND	ND	NG	NG	Yes (*C_T_* = 30.9)
SCHI0175-C	Monomicrobial (*Moraxella osloensis*)	ND	***Moraxella osloensis^e^***	NG	No (*C_T_* = 31.9)

^*a*^ Abbreviations: *C*_T_, cycles-to-threshold; MALDI-TOF, matrix-assisted laser desorption/ionization time-of-flight mass spectrometry; ND, not determined; NG, no growth; PE, pleural effusion. Bolded text denotes footnoted item; gray-shaded fields indicate discordant negative pleural infection diagnosis.

^*b*^ Minimum read threshold of ≥400 reads.

^*c*^ Minimum read threshold of ≥40 reads.

^*d*^ Based on TRIzol interphase DNA extraction.

^*e*^ Cultures subjected to whole-genome sequencing.

^*f*^ *Escherichia coli* excluded from the metagenomic output due to its read count being lower than the background (contaminant) signal.

^*g*^ No *S. saprophyticus* or other coagulase-negative staphylococci were identified, suggesting potential taxonomic misassignment or a detection limit issue.

^*h*^ *Pichia kudriavzevii* identified in the metagenome data (1,963 reads).

^*i*^ Probable taxonomic misassignment of certain “*Staphylococcus*” as “*Streptococcus*” using bacterial metataxonomics.

^*j*^ SCHI0131-P yielded exceptionally high and diverse microbial burden across all testing methods; this case differed clinically from others in that the participant had suffered a ruptured oesophagus (Boerhaave’s syndrome).

### qPCR performance

Positive bacterial detection was achieved with panbacterial qPCR in 14 out of 26 (54%) suspected pleural infection cases, including 12 out of 16 (75%) probable infection cases and 2 out of 10 (20%) possible infection cases (Table 3). All panbacterial qPCR-positive results were also bacteria-positive according to metagenomics and/or bacterial metataxonomics (Table 3). One Unlikely infection case (10%) was panbacterial qPCR-positive, albeit at a borderline *C_T_* value of 30.9; this result was not supported by any of the other tested methods. Panfungal qPCR was negative across all 36 specimens.

### Bacterial metataxonomic and shotgun metagenomic performance

**Fig 1:**
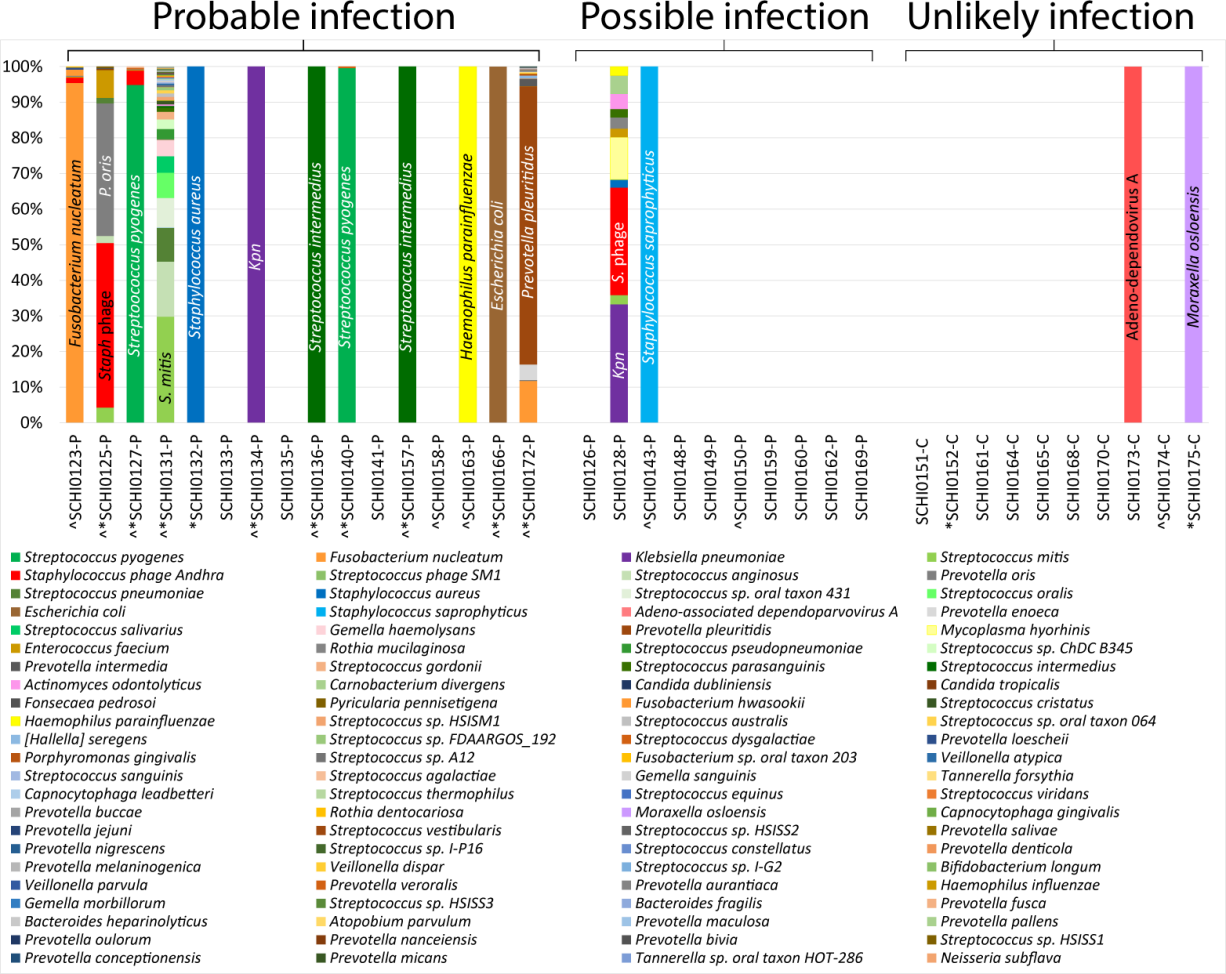
Metagenomic profiles identified from 26 patients with suspected pleural infection specimens and 10 Unlikely infection cohort specimens. *Conventional culture positive; ^16S ribosomal RNA (panbacterial) quantitative PCR positive; #Whole-genome sequencing-confirmed (from culture). Dominant taxa are labeled on each bar.

Bacterial metataxonomics and metagenomics together identified 13 out of 16 (81%) Probable and 3 (30%) Possible pleural infection cases as infection-positive, with 12 out of 16 (75%) Probable and 2 out of 10 (20%) Possible cases identified by each individual method. Metagenomics identified 7 out of 14 (50%) cases as polymicrobial, versus 5 out of 14 (36%) using bacterial metataxonomics (Table 3; Fig. 1). Despite both methods identifying 14 pleural infection cases each, only 12 overlapped, and only 3 of these cases were identified as polymicrobial by both methods (Table 3). Far fewer taxa were identified by bacterial metataxonomics in two of these three cases (SCHI0131-P and SCHI0172-P) (Table 3).

### Molecular diagnostics versus microbial culture performance

Compared with our Probable pleural infection-positive clinical reference classification, all molecular diagnostic methods demonstrated superior sensitivity and negative predictive values (NPVs) compared with microbial culture (Table 4), although all values (except positive predictive values [PPVs]) became much weaker when Probable and Possible pleural infection-positive cases were combined (Table S2). As expected, specificity and PPVs remained high across all diagnostic methods (Table 4).

**TABLE 4:** Comparative diagnostic accuracy of conventional culture and molecular methods for detecting pleural infection in Probable pleural infection cases^*a*^

	Conventional culture	Panbacterial qPCR	Bacterial metataxonomics	Metagenomics	Combined molecular
Sensitivity (95% CI)	62.5 (38.8–86.2)	75.0 (53.8–96.2)	75.0 (53.8–96.2)	75.0 (53.8–96.2)	81.2 (57.0–93.4)
Specificity (95% CI)	100 (100–100)	90.0 (71.4–100)	80.0 (55.2–100)	80.0 (55.2–100)	70.0 (39.7–89.2)
PPV (95% CI)	100 (100–100)	92.3 (77.8–100)	85.7 (67.4–100)	85.7 (67.4–100)	81.2 (57.0–93.4)
NPV (95% CI)	62.5 (38.8–86.2)	69.2 (44.1–94.3)	66.7 (40.0–93.3)	66.7 (40.0–93.3)	70.0 (39.7–89.2)

^*a*^ Abbreviations: CI, confidence interval; NPV, negative predictive value; PPV, positive predictive value; qPCR, quantitative PCR; Combined Molecular, all molecular methods (qPCR, bacterial metataxonomics, metagenomics).

^*b*^ Probable pleural infection: (i) the presence of Gram stain- and/or culture-positivity from pleural fluid according to Pathology laboratory testing or (ii) pleural fluid pH ≤7.2 and/or glucose <3.0 mmol/L, and (iii) a clinical presentation consistent with pleural infection including two or more of the following: fever (>38°C); raised inflammatory markers (serum C-reactive protein >100 mg/L or total peripheral blood white cell count (>11.0 × 10^9^ /L); complex pleural fluid on imaging.

**Fig 2:**
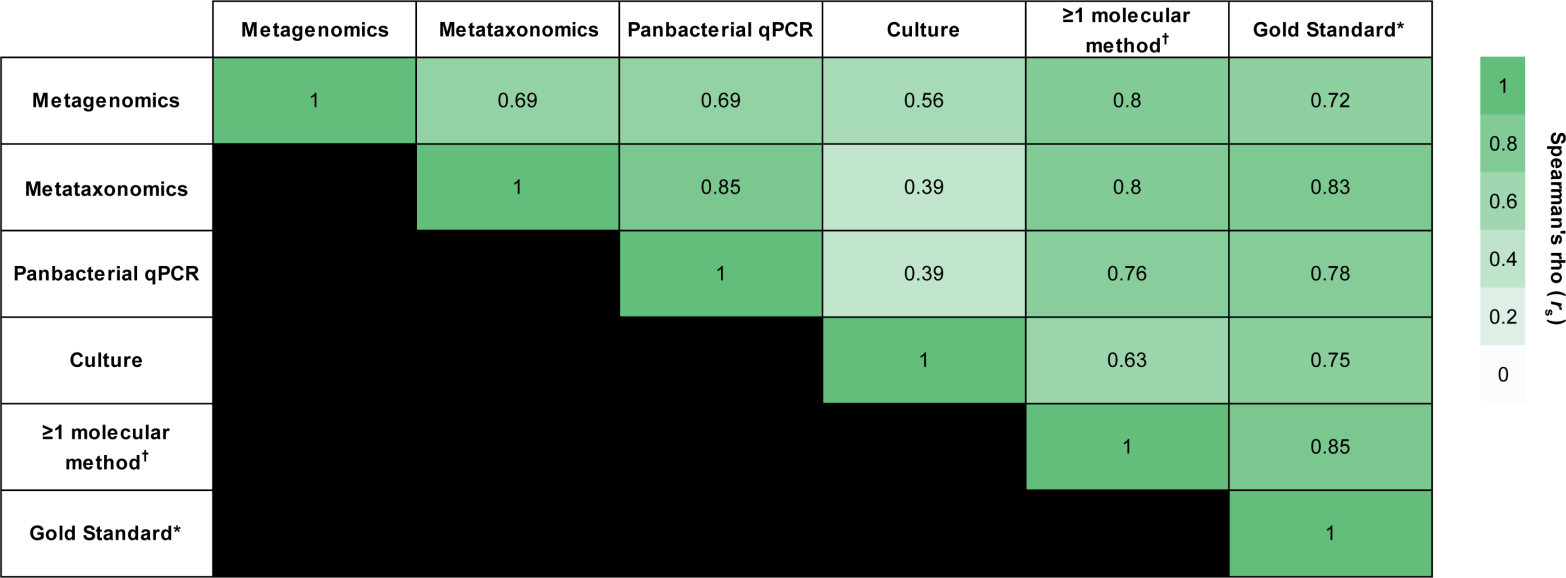
Correlation matrix of the pleural infection diagnostic methods used in this study. 1 = perfect positive correlation; 0 = no correlation; −1 = perfect negative correlation. Conventional culture showed the weakest correlation when compared with each of the three molecular methods.

Conventional culture somewhat correlated with molecular methods (metagenomics: *r_s_* = 0.56 [*P* < 0.001]; bacterial metataxonomics: *r_s_* = 0.39 [*P* = 0.05]; panbacterial qPCR: *r_s_* = 0.39 [*P* = 0.05]) (Fig. 2). Despite being derived from a different pleural fluid fraction to that used for bacterial metataxonomics and panbacterial qPCR, metagenomics demonstrated the strongest correlation across all methods (bacterial metataxonomics: *r_s_* = 0.69 [*P* < 0.001]; panbacterial qPCR: *r_s_* = 0.69 [*P* < 0.001]; and conventional culture: *r_s_* = 0.56 [*P* < 0.01]). The strongest individual correlation was between panbacterial qPCR and bacterial metataxonomics (*r_s_* = 0.85; *P* < 0.001; Fig. 2); this result was expected as both methods target the 16S rRNA gene and were tested from the same DNA extraction.

In 5 out of 26 suspected pleural infection specimens (SCHI0136-P, SCHI0140-P, SCHI0141-P, SCHI0143-P, and SCHI0166-P), we experienced delays (19–150 days) between specimen collection and Research lab processing. In two of these cases, *S. intermedius* (SCHI0136-P, 134 days), and *S. pyogenes* and *S. aureus* (SCHI0140-P, 35 days), were cultured by the Pathology lab, but not by the Research lab. SCHI0166, which had the longest processing delay (150 days) but the best panbacterial qPCR *C*_*T*_ value (21.5; Table 3) of the five specimens, cultured *E. coli* in both labs. In four out of five cases, metagenomics, bacterial metataxonomics, and qPCR showed 100% concordance, with only SCHI0136-P yielding a false-negative result using bacterial metataxonomics.

### Pleural infection etiology

**Fig 3:**
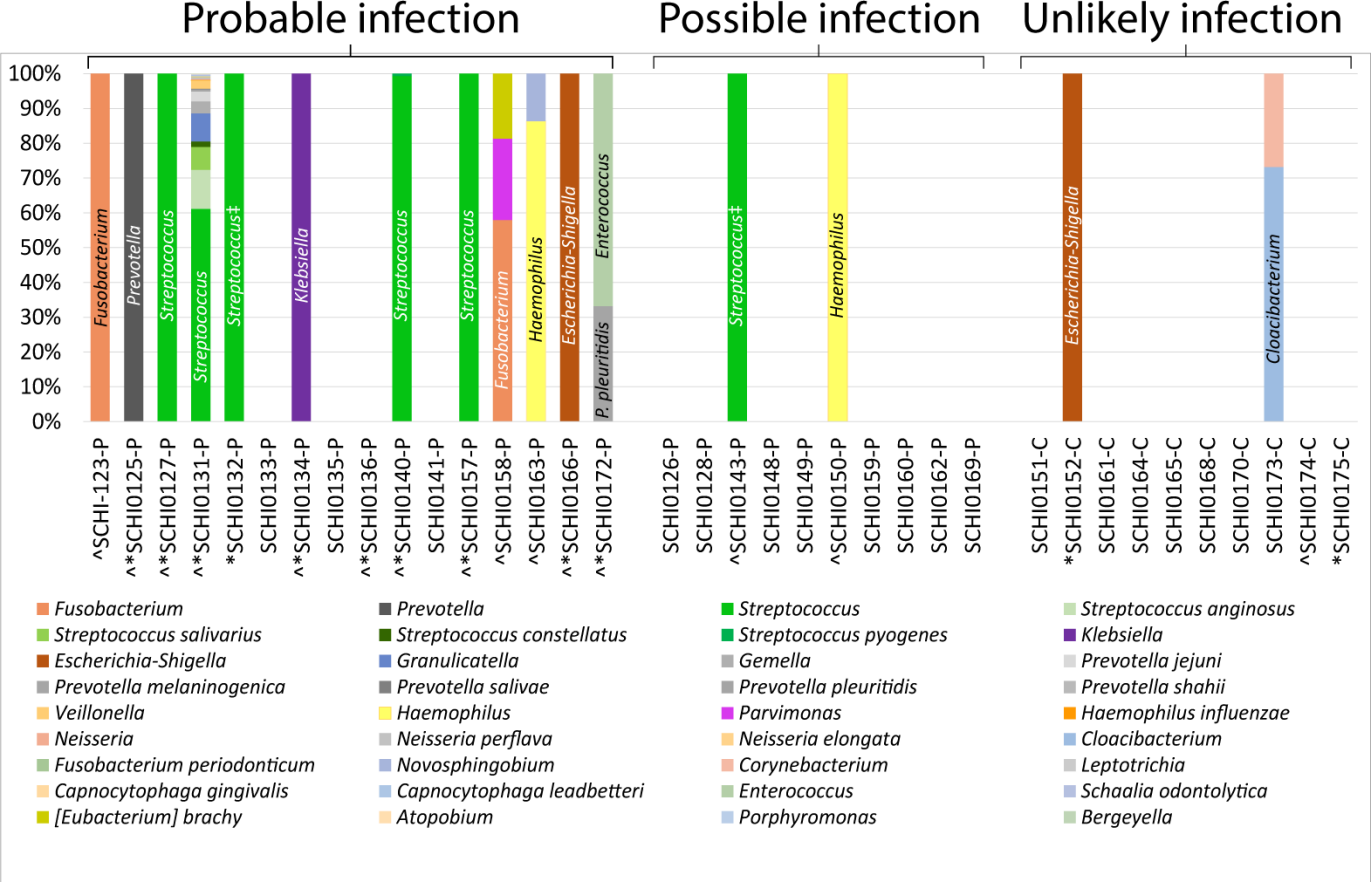
Bacterial 16S ribosomal RNA (rRNA) metataxonomic profiles identified from 26 suspected pleural infection specimens and 10 control cohort specimens. *Conventional culture positive; ^16S ribosomal RNA (panbacterial) quantitative PCR positive; #Whole-genome sequencing result from culture; ‡Likely incorrect assignment of *Staphylococcus* as *Streptococcus* (one case confirmed as a coagulase-negative *Staphylococcus* by MALDI-TOF analysis of culture). Dominant taxa are labeled on each bar.

A diverse array of oro-naso-hypopharyngeal-, oral-, respiratory tract-, and gut-associated microbes were detected in the 14 metagenomic infection-positive specimens (Table 3; Fig. 1). Streptococci (*n* = 5; 2 × *S*. *intermedius*, 2 × *S*. *pyogenes*, 1 × *S*. *mitis*) dominated, followed by *Prevotella* spp. (*n* = 2; *P. oris* and *P. pleuritidis*), *Staphylococcus* spp. (*n* = 2; *S. aureus* and *S. saprophyticus*), and *K. pneumoniae* (*n* = 2) (Table 3). Seven (50%) cases were monomicrobial according to metagenomics, of which two harbored *S. intermedius*, along with one each of *K. pneumoniae*, *S. saprophyticus*, *S. aureus*, *Enterococcus faecium*, and *E. coli* (Table 3; Fig. 3). In contrast, bacterial metataxonomics identified nine (35%) monomicrobial pleural infection cases, of which four were *Streptococcus* sp., along with one each of *Escherichia-Shigella* sp., *Fusobacterium* sp., *Haemophilus* sp., *Klebsiella* sp., and *Prevotella* sp. (Table 3; Fig. 3). Five monomicrobial cases overlapped with the two meta-omics methods; however, bacterial metataxonomics erroneously assigned two monomicrobial pleural infection cases, SCHI0132-P and SCHI0143-P, as “*Streptococcus*,” despite the SCHI0132-P isolate being confirmed as coagulase-negative *Staphylococcus* sp. by VITEK 2 (Table 3), indicating a probable bacterial metataxonomic database error for this genus.

As expected, bacterial metataxonomics failed to identify fungi or viruses in the pleural infection cases, whereas conventional culture and metagenomics identified the yeast, *Pichia kudriavzevii*, in SCHI0131-P. Metagenomics further identified the fungi *Rhizoctonia solani*, *Wallemia mellicola*, *Meyerozyma guilliermondii*, *Truncatella angustata*, *Cokeromyces recurvatus*, *Bipolaris maydis*, *Candida orthopsilosis*, and *Gilbertella persicaria* in SCHI0131-P (subject suffered from confirmed Boerhaave’s syndrome), *Candida dubliniensis* in SCHI0140-P, and *Candida tropicalis*, *Fonsecaea pedrosoi*, and *Pyricularia pennisetigena* in SCHI0125-P. In all instances, fungi were in relatively low abundance. Metagenomics also identified bacteriophages in 5 out of 26 infection-positive cases, with *Staphylococcus* phage Andhra seen in SCHI0123-P, SCHI0125-P, SCHI0127-P, and SCHI0128-P, and *Streptococcus* phage SM1 seen in SCHI0131-P. *Staphylococcus* phage Andhra was among the most dominant taxon seen in SCHI0125-P and SCHI0128-P, indicating very high phage levels in pleural fluid; both specimens harbored *S. aureus* according to metagenomics, and SCHI0125-P also had *S. saprophyticus* according to culture and whole-genome sequencing (WGS) (Table 3; Fig. 3).

Metagenomics unexpectedly found microbes in 2 out of 10 Unlikely infection cases, both of whom had MPE (Table 3). In the first case, the non-pathogenic DNA virus, adeno-associated dependoparvovirus A, was identified in SCHI0173-C, although no helper virus was observed. In the second case, *M. osloensis* was identified in SCHI0175-C; this bacterium was cultured-confirmed in the Research lab, but not the Pathology lab. In contrast, *C. parapsilosis* cultured from SCHI0152-C in the Research lab only was not detected by metagenomics, suggesting possible operator contamination during specimen collection or Research lab processing. Bacterial metataxonomics failed to identify the culture-confirmed *M. osloensis* in SCHI0175-C, but identified *Escherichia-Shigella* in SCHI0152-C (1453 reads) and *Cloacibacterium* and *Corynebacterium* in SCHI0173-C (519 and 190 reads, respectively), findings that were not supported by the other methods.

### Microbiology and mortality

Of the three pleural infection cases with <30-day mortality, two (SCHI0128-P and SCHI0134-P) were infected with *K. pneumoniae*, and two (SCHI0131-P and SCHI0128-P) had polymicrobial infections according to metagenomics. In contrast, no patient surviving beyond 30 days (*n* = 23) harbored *Klebsiella* spp. according to any testing method.

### Association between clinical biomarkers and pleural infection

Comparison of biochemical markers (Table S1) between the Probable and Possible pleural infection cohorts identified significant differences in total white cell count (*P* = 0.009), pleural fluid pH (*P* < 0.001), and pleural fluid glucose (*P* < 0.001), consistent with the inclusion of these biomarkers in the clinical diagnostic criteria. However, when comparing Probable and Possible infection participants who had culture- or molecular-confirmed infections with their counterparts lacking culture or molecular evidence of infection, only pleural fluid glucose (*P* = 0.004) was significantly different between these two groups.

### AMR gene detection

Only innate drug resistance genes were identified, with no evidence of horizontally acquired AMR in any case (Table 1).

## DISCUSSION

Clinical decision-making in suspected pleural infection is challenging due to low culture positivity rates (5, 12, 21, 22), resulting in frequent diagnostic uncertainty and antimicrobial overuse. Recent work (5, 11–13, 16, 19) has highlighted the potential advantages of molecular methods for diagnosing and determining pleural infection etiology. Our pilot feasibility study adds to this growing body of work, being the fourth study to date to have applied shotgun metagenomics on pleural infections, and the first to prospectively collect and profile consecutive pleural fluid specimens.

In line with recent molecular studies (5, 11–14), we confirm that pleural infections can be either monomicrobial or polymicrobial, with predominant microbes most likely originating from oral/dental or upper and lower respiratory tract sources, and occasional incursion from gut-borne pathogens (e.g., *E. coli*, *E. faecium*, and *K. pneumoniae*) (Table 3). Furthermore, metagenomics and bacterial metataxonomics unveiled greater microbial heterogeneity than could be detected by conventional culture. Similar to recent work (13, 16), we observed that polymicrobial bacterial infection was also occasionally associated with fungal coinfection (albeit in very low abundance) using metagenomics, confirming the challenging nature of diagnosing and treating complex polymicrobial pleural infections across microbial domains. Streptococcal and staphylococcal phages were also seen in five cases of pleural infection, with their host species not always detected, suggesting possible lytic activity toward their host within the pleural space, or incidental phage transit from other body sites (23). Although not examined here, others have shown that phage presence in clinical specimens can negatively impact culture rates (24). Despite our low sample numbers, we found that *K. pneumoniae* presence was potentially associated with greater risk of mortality, with two out of three fatal cases in our study harboring this pathogen. This observation is consistent with a recent bacterial metataxonomic study that showed an association between Enterobacteriaceae and mortality (5); high mortality rates have also been reported in alcoholics with bacteremic *K. pneumoniae* pneumonia and co-morbid pleural effusion (25). Notably, metagenomics was the only method that identified *K. pneumoniae* in these two fatal cases.

Immediate antimicrobial administration upon suspicion of pleural infection, which often occurs prior to pleural fluid collection, is important for preventing sepsis (26); however, this practice may negatively affect pleural infection diagnostic rates. Despite this, many prior pleural microbiome studies have identified 100% infection positivity using molecular testing (5, 11, 12). In contrast, only 81% of Probable pleural infections and 30% of Possible pleural infections in our study (i.e., overall positivity rate of 62% across both Probable and Possible infection cohorts) had detectable microbes according to molecular and/or culture assessment. Our lower positivity rate likely reflects our prospective, consecutive recruitment of participants without prior knowledge of culture results or pleural biochemistry, an approach that captures the diagnostic uncertainty facing clinicians in real-world practice. Only two other studies have not used prior knowledge of pleural fluid biochemistry or microbiology for molecular pleural infection identification and characterization; their infection positivity rate was 70% (13, 14). Our finding that 38% of Probable and Possible pleural infection participants did not have detectable microbes according to any method suggests that a subset may instead have sterile pleural effusions at the time of sampling. In support of this conclusion, these patients had significantly higher (*P* = 0.004) pleural fluid glucose levels (range: 2.4–11.9 mmol/L, median: 4.4 mmol/L) than their counterparts with a culture- or molecular-confirmed infection (range: 0.3–5.9 mmol/L, median: 0.3 mmol/L). A second possibility is that some infections were missed due to recent antibiotic exposure that preceded their pleural effusion event; however, others have shown that molecular approaches are less impacted by prior antibiotics than conventional culture (12, 13). In addition, we excluded patients who had received antimicrobial therapy up to three weeks prior to pleural sampling, and did not observe any acquired AMR determinants in any enrolled participant (Table 1), suggesting minimal impact of prior antimicrobial administration on the pleural microbiome in our cohort. A third possibility is that human DNA depletion prior to metagenomic sequencing may have limited our ability to detect extracellular microbial DNA (27). To overcome this issue, deeper metagenomic sequencing on total DNA can be conducted; however, such an approach is costly, and risks missing low-abundance microbes due to overwhelming human DNA signal (27, 28). Prospective randomized trials are needed to understand whether a truncated antibiotic course can be safely used in people with likely sterile effusions as identified with molecular diagnostics, and to quantify the impact of prior antibiotic administration on culture or molecular detection sensitivity.

Sensitive molecular assays come with the trade-off that microorganism presence might represent either a contaminant or a bona fide pathogen. To assess this issue, we investigated whether the Unlikely infection group, predominantly comprised of MPEs, had a pleural microbiome. Although most Unlikely infection cases had a sterile pleural space, one MPE patient, SCHI0175-C, was positive for *M. osloensis* upon Research lab culture, WGS, and metagenomics, although not on bacterial metataxonomics or Pathology lab culture. *M. osloensis*, a commensal of human skin and the upper respiratory tract, most commonly causes infection in cancer patients (29); it is thus likely that this patient had an undiagnosed infection alongside their MPE. Three other MPE patients also unexpectedly yielded microbes: *C. parapsilosis* was cultured from SCHI0152-C in the Research lab, and bacterial metataxonomics identified *Corynebacterium* and *Escherichia-Shigella* in SCHI0173-C and SCHI0152-C, respectively; however, these findings were not supported by the other methods. Although specimen or sampling contamination represents the most plausible explanation for these results, a recent bacterial metataxonomic study suggested that non-infective pleural disease may contain unique microbial signatures (12, 30). Strict aseptic practice during specimen collection and processing, and sequencing larger numbers of reagent-only controls, might mitigate some of these false-positive cases. However, microbial contamination may still occur at many stages of sample handling and analysis, including procedural contamination (e.g., remnant microbial DNA in the specimen jars or local anesthetics), or residual DNA from transient microbial seeding of the pleura. Careful consideration of these challenges will be important in the design of larger prospective studies. Further studies examining the pleural microbiome across a spectrum of pleural diseases, including in people with MPE and congestive heart failure, will also be illuminating.

Panbacterial qPCR stands out as a cost-effective, simple, and same-day diagnostic that could be readily implemented as a routine test for pleural fluid specimens; such a test may assist with both antimicrobial stewardship efforts and rapid treatment decision-making (31). In support of its clinical utility, panbacterial qPCR showed excellent diagnostic performance (sensitivity 82%, specificity 95%, PPV 93%, and NPV 86%) and strong correlation with shotgun metagenomics, bacterial metataxonomics, and conventional culture. Although not evaluated in this study, the use of panbacterial digital PCR may further improve suspect pleural infection diagnoses due to the higher precision associated with this method, especially in low-biomass specimens (32).

This study has several recognized limitations. Although the first study to systematically compare performance across these diagnostic methods, we acknowledge our relatively small sample size. Expanding this proof-of-principle work on a larger scale, and across multiple testing sites, will be important for understanding the diagnostic performance and clinical utility of these tests among broader Australian and international cohorts. Index hopping and “kitome” contamination are known drawbacks of high-throughput sequencing, especially in low-biomass specimens (28), and are typically controlled for by subtracting background signals from specimen data. Although this approach is common, it is likely not sufficient for removing all contaminating DNA (33), and risks removing real microbial signal. Indeed, this approach resulted in a culture-confirmed *E. coli* infection being omitted in SCHI0125-P due to higher *E. coli* metagenomic and *Shigella-Escherichia* bacterial metataxonomic reads in the reagent-only controls (Table 4). One way to reduce this issue might be to employ ultraclean, certified microbe-DNA-free reagents (34), although using such reagents represents an added cost. “Incorporation bias” (35) may have inflated the performance of conventional culture results, given that this method contributed to our diagnostic reference standard. We did not assess RNA viruses in this study using viral-specific qPCRs or metatranscriptomics. However, to our knowledge, this technique has not yet been undertaken to investigate pleural infections, so its value in this cohort remains unclear. Despite the promise of molecular diagnostic methods, there remains uncertainty regarding microbial causality and pathogenicity (e.g., the role of bystander microbes in pleural disease development and prognosis), particularly in polymicrobial infections. As our study was prospective, we did not assess the impact of specimen freeze-thawing on microbial DNA detection capability. We did not undertake dideoxy sequencing of panbacterial qPCR amplicons, particularly those generated at higher *C*_*T*_ values, to determine whether they were caused by a suspect etiologic agent versus background signal; doing so may have lowered our panbacterial *C*_*T*_ cutoff. Perhaps most challenging is the current lack of gold-standard clinical diagnostic criteria for pleural infection. In clinical practice, an integrated approach is taken to weigh multiple factors to make a probabilistic diagnosis, including pleural biochemistry, host response, and imaging. In this study, we attempted to standardize integrated probabilistic diagnostic classification by formulating a pragmatic reference standard to compare diagnostic performance between modalities. However, the issue of a validated diagnostic gold standard remains an unresolved challenge.

We experienced specimen processing delays in five suspected pleural infection cases that may have impacted Research lab microbial cultivability in two of these cases (SCHI0140-P and SCHI0136-P). However, these delays did not demonstrably impact molecular findings, with strong concordance in four out of five cases, and no overt taxonomic differences compared with Pathology lab culture. In just one case, SCHI0136-P (134-day delay), bacterial metataxonomics yielded a false-negative result compared with the other methods, despite being generated from the same DNA sample as the panbacterial qPCR result (*C*_*T*_ = 27.5; Table 1). Our findings suggest that all three culture-independent molecular techniques tested in our study—metagenomics, bacterial metataxonomics, and panbacterial qPCR—have good performance characteristics for pleural infection diagnosis, and may be superior to conventional culture for diagnosis and etiologic confirmation, particularly when there are delays between specimen collection and processing. This observation warrants further exploration.

In conclusion, this prospective study demonstrates the feasibility of, and challenges associated with, molecular tests for pleural infection diagnosis. While such methods hold great clinical utility and potential superiority in routine pleural infection diagnostics, questions and challenges remain. A large, rigorous study comparing molecular and culture-based diagnostic techniques in pleural infection is now required to further address these opportunities for diagnostic advancement, along with the associated challenges in eventual clinical translation.

## MATERIALS AND METHODS

### Study participants

All patients ≥18 years of age presenting with suspected pleural infection (denoted with a “-P” suffix) admitted between March and November 2022 to the Sunshine Coast University Hospital (SCUH), QLD, Australia, and undergoing a diagnostic and/or therapeutic procedure for suspected pleural infection, were prospectively invited.

Due to the lack of a gold standard pleural infection confirmation test, “clinical likelihood of pleural infection” was objectively stratified by three specialist respiratory physicians (PTB, TB, and JG), using three predefined clinical criteria: *1. Probable pleural infection*: (i) the presence of Gram stain- and/or culture-positivity from pleural fluid according to Pathology laboratory testing or (ii) pleural fluid pH ≤7.2 and/or glucose <3.0 mmol/L, and (iii) a clinical presentation consistent with pleural infection including two or more of the following: fever (>38°C); raised inflammatory markers (serum C-reactive protein >100 mg/L or total peripheral blood white cell count >11.0 × 10^9^ /L); complex pleural fluid on imaging; *2. Possible pleural infection*: (i) pleural fluid that is Gram stain- or culture-negative with pH ≥7.2 and/or glucose >3.0 mmol/L and (ii) one or more of the following: fever (>38°C); serum C-reactive protein >100 mg/L; complex pleural fluid on imaging; *3. Unlikely pleural infection*: (i) Clinical presentation not consistent with infection (as defined in (iii) above), and (ii) all of the following criteria are met: pleural fluid is Gram stain- or culture-negative, has a pH ≥7.2, glucose levels of >3 mmol/L, uncomplicated effusion on computed tomography or ultrasound imaging; and a confirmed alternative diagnosis (i.e., heart failure and malignancy). These criteria are similar, but not identical, to those previously reported in the PILOT (36) and TORPID (5) studies. Based on these criteria, 26 consecutive patients with Probable or Possible pleural infections (denoted with a “-P” suffix), and 10 consecutive patients in the Unlikely category (denoted with a “-C” suffix), were enrolled. Patients in any group were excluded if they had prior sampling of the pleural space, any clinical concern for current or recent infection, concomitant abdominal ascites, hepatic hydrothorax, or antibiotic administration within 3 weeks of pleural sampling.

### Specimen collection

The study Research and Pathology teams were provided participant categorization (“-P” vs “-C”) by the study clinicians, but no other information. For the pleural infection cohort, specimens were collected as soon as practicable using aseptic technique. Antibiotic commencement ranged from 21 days prior to pleural sampling to immediately post-pleural aspiration (Table 1). Following 10 mL of 1% lidocaine subcutaneous administration, 10–60 mL of pleural fluid was collected into two sterile specimen pots. One pot was immediately mixed with 7 mL stabilization agent (37) for total DNA extraction (bacterial metataxonomics and qPCR); the second pot contained only pleural fluid for culture and host DNA depletion and extraction (metagenomics). Specimens were immediately stored at 4°C until processed. For the Unlikely infection cohort, specimens were stored for between 0 and 5 days prior to processing. For the suspected pleural infection cohort, 21 out of 26 specimens were stored for between 0 and 14 days prior to processing; the remaining five specimens were stored for 19 (SCHI0143-P), 21 (SCHI0141-P), 35 (SCHI0140-P), 134 (SCHI0136-P), and 150 (SCHI0166-P) days prior to processing. In parallel, specimens were also collected for routine microscopy (cell count, white blood cell differential, and Gram stain), and biochemistry analyses (pleural fluid pH, protein, glucose, lactate dehydrogenase, and serum C-reactive protein).

### Microbiological culture

Culturing was independently undertaken by both the Research and Pathology lab teams. The Pathology workflow involved: (i) inoculating ~10 mL pleural fluid into anaerobic and aerobic blood culture bottles, which were incubated at 37°C for up to 5 days in a blood culture incubator (BacT/ALERT 3D, bioMérieux, North Ryde, NSW, Australia), and (ii) direct plating of pleural fluid onto horse blood and chocolate agars, followed by 37°C incubation in 5% CO_2_ or anaerobic atmospheres for 5 days, with daily inspection for culture growth. All culture-positive specimens were subjected to VITEK MS (bioMérieux, NSW, Australia) matrix-assisted laser desorption/ionization time-of-flight mass spectrometry to confirm species identity.

The Research lab workflow involved subjecting the pleural fluid to an initial centrifugation step of 290 × *g* at 4°C for 15 min to pellet gross cellular material (Pellet 1), as previously described (12). The supernatant was transferred to a fresh tube and subjected to a second centrifugation step of 9,200 × *g* at 4°C for 15 min to pellet the remaining cellular material (Pellet 2). Approximately 20 µL of the resuspended pellets (for all specimens) and supernatant (for the first 10 specimens only) were subjected to heat soak DNA extraction using 5% chelex 100 (38) resin (Bio-Rad, South Granville, NSW, Australia) for 10–20 min at 95°C. Following resin pelleting at 10,000 × *g* for 1 min, supernatant was diluted 1:10 with water and used for qPCR (see qPCR Testing below). Following chelex qPCR testing, 2× ~50 µL samples of the best-performing fraction/s (Pellet 1, Pellet 2, or supernatant) were glycerol-stocked, and ~20 µL specimen was plated and 16-streaked onto various agars (MacConkey, Sabouraud dextrose, mannitol salt, Columbia horse blood, and supplemented chocolate [Edwards Group, Narellan, NSW, Australia]) and incubated aerobically at 37°C to isolate common pathogens and oral/airway microbes. Blood and chocolate agars were also inoculated for anaerobic incubation in an Anaerobox jar using AnaeroPouch anaerobic gas generators (Thermo Fisher Scientific, Scoresby, VIC, Australia), with anaerobic atmosphere confirmed via resazurin anaerobic indicator strips (Thermo Fisher Scientific). Culture growth was assessed at 24 h and then periodically for 7 days. Plates lacking any observable growth after 7 days were deemed as “no growth.” A subset of culture-positive specimens was subjected to Illumina NovaSeq 6000 WGS (Australian Centre for Ecogenomics, QLD, Australia) to confirm species identity.

### DNA extraction for bacterial metataxonomics

The stabilization reagent specimens (excluding SCHI0123-P and SCHI0125-P) were centrifuged at 9,200 × *g* and 4°C for 15 min to pellet all cellular material; the supernatant was decanted, followed by 10 parts addition of TRI Reagent and 30 µL β-mercaptoethanol (Sigma-Aldrich) to the pellet to inactivate RNases. For SCHI0123-P and SCHI0125-P, Pellet 2 from the pleural fluid pot was used for TRI Reagent DNA extraction due to no stabilization reagent pot being collected for these specimens. DNA was retrieved from the interphase/proteinaceous phase using the back extraction buffer method (39); the aqueous phase was removed and stored at −80°C for future RNA extraction. Three reagent-only controls were also processed.

### DNA extraction for metagenomics and WGS

Pleural fluid was human DNA-depleted using the “Benzonase 1” method (27). Cells were then lysed using an in-house metapolyzyme concoction containing 50 U mutanolysin, 280 KU lysozyme, 4.4 U lysostaphin, 0.03 U chitinase, 10 U achromopeptidase (Sigma-Aldrich, Bayswater, VIC, Australia), and 60 U zymolyase (MP Biomedicals, Irvine, CA, USA), which was incubated in 200 µL enzymatic lysis buffer (1.2% Triton-X, 2× Tris-EDTA, pH = 8.0) at 37°C for 2 h. DNA extraction was carried out using the Gram-Positive Bacteria protocol from the DNeasy Blood & Tissue kit (Qiagen, Clayton, VIC, Australia). Seven reagent-only controls were included to account for background DNA contamination in extraction and sequencing reagents. Bacterial and fungal cultures for WGS were extracted as above, but without benzonase depletion.

### Bacterial metataxonomics

All 36 pleural fluid specimens were subjected to 16S rRNA V3-V4 amplification using Bakt_341f and Bakt_805r primers (40), followed by Illumina MiSeq 300 bp paired-end sequencing (Ramaciotti Centre for Genomics, Sydney, NSW, Australia). Illumina adapters were demultiplexed and trimmed with QIIME 2 (41) using a *P* error rate of 0.20. DADA2 (42) was then used to denoise, quality-filter (forward and reverse reads trimmed to 261 and 199 bp, respectively), and assign amplicon sequence variants for taxonomic assignment using the silva-138-99-nb-classifier (43). A minimum cutoff of 40 reads was used to define microbial presence. Where at least one of the three reagent-only controls had a given taxon exceeding 40 reads, all specimen data sets had this equivalent signal manually removed from the tabulated barchart output. Only taxon assignments at the genus or genus-species level were recorded; phyla through Family levels were discarded.

### Metagenomic sequencing

Illumina 150 bp paired-end reads (16–28 million reads/sample) were generated from all 36 host DNA-depleted specimens using the NovaSeq 6000 platform (17–27 million reads/sample; Azenta Life Sciences, Suzhou, China), resulting in between 17 and 27 million reads per sample. Seven reagent-only controls were also sequenced to account for background microbial signal and index hopping. Metagenomic reads were first quality-filtered with Trimmomatic v0.39 (44) using TruSeq Illumina adapter removal, leading = 3, trailing = 3, sliding window = 4 :15, and minimum length = 36 (45). Next, human reads were identified using the Kraken 2 v2.1.2 (46) human database, followed by removal using Seqtk v1.3 (https://github.com/lh3/seqtk). Non-human reads were assigned microbial taxonomies by interrogation against the default full Kraken 2 bacterial, fungal and viral database, with the fungal database expanded to include genomes of chromosome, scaffold, and contig completeness. Contaminant signal, identified both from reagent-only controls and by manually assessing the ecology of each microbe, was removed using Seqtk. Microbes with higher abundance in one or more clinical specimens compared with reagent-only controls were retained. A minimum cutoff of 400 reads was used to define microbial presence in the clinical specimen metagenomic data.

### Whole-genome sequencing

To confirm species identity, Illumina 100 bp paired-end reads (34–496×) were generated for five cultures (SCHI0125.M.1, SCHI0125.M.2, SCHI0131.M.3, SCHI0152.S.1, and SCHI0175.S.1) retrieved from two Probable pleural infection participants (SCHI0125-P and SCHI0131-P) and Unlikely infection participants (SCHI0152-C and SCHI0175-C) using the NovaSeq 6000 platform (Australian Centre for Ecogenomics, St Lucia, QLD, Australia). Genomes were quality-filtered with Trimmomatic using the same parameters as the metagenome sequences, followed by *de novo* assembly with SPAdes v3.15.5 (47).

### qPCR testing

Total genomic DNA was tested against panbacterial (16S rRNA) (48), panfungal (28S rRNA) (49), and human (β-globin) (50) qPCR assays to quantify microbial-to-host load. For the panbacterial qPCR assay, a cycles-to-threshold (*C*_*T*_) cutoff value of 31 was used to delineate pleural infection-positive versus -negative cases; this cutoff represents the highest *C*_*T*_ value not observed in any of the reagent-only controls. For the panbacterial and human qPCR assays, we omitted the probe and instead ran as SYBR Green formats. For the panbacterial and panfungal assays, we used 0.3 and 0.2 µM each primer, respectively. All three assays were run with the following thermocycling parameters: 3 min initial denaturation at 95°C, followed by 45 cycles of 95°C for 10 s and 60°C for 20 s. All qPCRs were run on a CFX96 Touch real-time PCR instrument (Bio-Rad) using 5 µL total reaction volumes and SsoAdvanced Universal SYBR Green supermix (Bio-Rad). All samples were run in duplicate, and all runs included positive and reagent-only controls.

### AMR gene assessment

Human-depleted metagenomic reads were examined for AMR gene presence using ResFinder v4.1.11 (51). AMR genes present in reagent-only controls or metagenomic-negative samples, and exhibiting <100% identity, were excluded.

### Statistical analyses

To determine diagnostic test performance, “Probable pleural infection” was used as the infection-positive reference. Specificity, sensitivity, PPV, and NPV were calculated for microbial culture, qPCR, bacterial metataxonomics, and metagenomics. Spearman’s rank correlation (*r_s_*) was used to determine pleural infection/no infection diagnostic correlations between the four testing methods. Unpaired *t* testing was used to determine associations between clinical biomarkers and pleural infection; a *P* value of ≤0.01 was considered significant.

## Data Availability

Raw bacterial 16S rRNA metataxonomic sequencing reads are available at NCBI BioProject PRJNA972883. Human-depleted metagenomic reads are available at BioProject PRJNA972883. Assemblies are available via BioProject PRJNA970939.

## SUPPLEMENTAL MATERIAL

Table S1 spectrum.01960-24-s0001.docx Clinical and demographic data for all participants examined in this study.

TABLE S2 spectrum.01960-24-s0002.docx Comparative diagnostic accuracy of conventional culture and molecular methods.

ASM does not own the copyrights to Supplemental Material that may be linked to, or accessed through, an article. The authors have granted ASM a non-exclusive, world-wide license to publish the Supplemental Material files. Please contact the corresponding author directly for reuse.
