# Supplementary material for: Evaluating the feasibility, sensitivity, and specificity of next-generation molecular methods for pleural infection diagnosis
Source: Microbiol Spectr. 2025 Jan 15;13(2):e01960-24. doi: 10.1128/spectrum.01960-24 (PMC11792517; doi:10.1128/spectrum.01960-24)
Supplement: TABLE S2 — Comparative diagnostic accuracy of conventional culture and molecular methods. [file spectrum.01960-24-s0002.docx]

**Table S2. Comparative diagnostic accuracy of conventional culture and molecular methods for detecting pleural infection in Probable and Possible* pleural infection cases.**

|  | Conventional culture | Panbacterial qPCR | Bacterial metataxonomics | Metagenomics | Combined molecular |
| --- | --- | --- | --- | --- | --- |
| Sensitivity (95% CI) | 38.5 (22.4-57.5) | 53.8 (35.5-71.2) | 53.8 (35.5-71.2) | 53.8 (35.5-71.2) | 61.5 (42.5-77.6) |
| Specificity (95% CI) | 100 (72.2-100) | 90.0 (59.6-98.2) | 80.0 (49.0-94.3) | 80.0 (49.0-94.3) | 70.0 (39.7-89.2) |
| PPV  (95% CI) | 100 (72.2-100) | 93.3 (70.2-98.8) | 87.5 (64.0-96.5) | 87.5 (64.0-96.5) | 84.2 (62.4-94.5) |
| NPV  (95% CI) | 38.5 (22.4-57.5) | 42.9 (24.5-63.5) | 40.0 (21.9-61.3) | 40.0 (21.9-61.3) | 41.2 (21.6-64.0) |

*Abbreviations:* CI, confidence interval; NPV, negative predictive value; PPV, positive predictive value; qPCR, quantitative PCR; Combined Molecular, all molecular methods (qPCR, bacterial metataxonomics, metagenomics). ****Probable pleural infection:*** (i) the presence of Gram stain- and/or culture-positivity from pleural fluid according to Pathology laboratory testing OR (ii) pleural fluid pH ≤7.2 and/or glucose <3.0 mmol/L, AND (iii) a clinical presentation consistent with pleural infection including two or more of the following: fever (>38^o^C); raised inflammatory markers (serum C-reactive protein >100 mg/L OR total peripheral blood white cell count >11.0 x 10^9^/L); complex pleural fluid on imaging; ***Possible pleural infection:*** (i) pleural fluid that is Gram stain- or culture-negative with pH ≥7.2 and/or glucose >3.0mmol/L AND (ii) one or more of the following: fever (>38^o^C); serum C-reactive protein >100 mg/L; complex pleural fluid on imaging.
